# Usage Trends and Data Sharing Practices of Healthcare Wearable Devices Among US Adults: Cross-Sectional Study

**DOI:** 10.2196/63879

**Published:** 2025-02-21

**Authors:** Ranganathan Chandrasekaran, Muhammed Sadiq T, Evangelos Moustakas

**Affiliations:** 1 Department of Information & Decision Sciences / Department of Biomedical and Health Information Sciences University of Illinois at Chicago Chicago, IL United States; 2 Department of Management Studies Indian Institute of Technology Madras Chennai India; 3 Department of Communication and Media Faculty of Communication, Arts and Sciences Canadian University of Dubai Dubai United Arab Emirates

**Keywords:** healthcare wearable devices, data-sharing behavior, willingness to share wearable data, activity trackers, wearable use, post-pandemic, wearables, healthcare delivery, disease detection, patient engagement, digital literacy, adults, United States, survey, cross-sectional survey, data sharing

## Abstract

**Background:**

Health care wearable devices can transform health care delivery by enabling real-time, continuous monitoring that facilitates early disease detection, personalized treatments, and improved patient engagement. The COVID-19 pandemic has heightened awareness of the importance of health technology, accelerating interest in wearables as tools for monitoring health and managing chronic conditions. As we navigate the postpandemic era, understanding the adoption and data-sharing behaviors associated with wearable devices has become increasingly critical. Despite their potential, challenges and low adoption rates persist, with significant gaps in understanding the impact of sociodemographic factors, health conditions, and digital literacy on the use and data-sharing behaviors of these devices.

**Objective:**

This study aimed to explore the usage and data-sharing practices (willingness to share wearable data and actual data-sharing behavior) of wearable devices among US adults specifically during the later phases of the COVID-19 pandemic.

**Methods:**

Using cross-sectional data from the National Cancer Institute’s Health Information National Trends Survey 6, conducted from March to November 2022, this study uses responses from 5591 US adults to examine wearable use, willingness to share wearable data with providers, family, and friends, and the wearable data-sharing behavior.

**Results:**

The results indicate an increase in wearable device adoption to 36.36% (2033/5591) in 2022, up from 28%-30% in 2019. We also find a significant discrepancy between the willingness to share data, with 78.4% (1584/2020) of users open to sharing with health care providers, and the actual sharing behavior, where only 26.5% (535/ 2020) have done so. Higher odds of using wearables were associated with female gender (odds ratio [OR] 1.49, 95% CI 1.17-1.90, *P*<.01) and higher income levels (OR 2.65, 95% CI 1.42-4.93, *P*<.01 for incomes between US $50,000 and US $75,000, and OR 3.2, 95% CI 1.71-5.97, *P*<.01 for incomes above US $75,000). However, the likelihood of usage and data sharing declines significantly with age. Compared with African American respondents, Hispanic respondents were more willing to share wearable data with providers (OR 1.92, 95% CI 1.02-3.62, *P*<.05), though the odds of their actual sharing of wearable data with providers was relatively less (OR 0.44, 95% CI 0.20-0.97, *P*<.05). Frequency of provider visits (OR 1.23, 95% CI 1.08-1.39, *P*<.01), and total medical conditions (OR 1.35, 95% CI 1.05-1.73, *P*<.01) were significant predictors of data-sharing behavior. The study also identified weight, frequency of provider visits, technological self-efficacy and frequent physical activity as predictors for higher wearable use.

**Conclusions:**

Insights from this study are crucial for health care providers and policy makers aiming to leverage wearable technology to enhance health outcomes. Addressing the disparities and barriers identified can lead to more effective integration of these technologies in health care systems, thereby maximizing the potential of digital health tools to improve public health outcomes.

## Introduction

Health care wearable devices, encompassing a diverse array of technologies ranging from smartwatches, activity trackers, to biosensors, are revolutionizing the landscape of health care delivery [1]. These devices, which include skin-based wearables, biofluidic-based wearables, and drug delivery systems [2], are typically worn on or close to the body, gather physiological data, and provide actionable insights into an individual’s health and well-being. By continuously monitoring vital signs, activity levels, and other biometric parameters, health care wearables offer opportunities for early disease detection, personalized treatment, and remote patient monitoring [3-5]. The potential of these devices to transform health care delivery is underscored by their ability to empower individuals to take proactive measures toward their health [6-8] and to facilitate health care professionals in delivering more personalized and timely interventions [9-11]. Furthermore, the integration of health data generated from wearable devices with mobile apps enhances their use, allowing for real-time tracking, monitoring, and analysis of health metrics [12]. With advancements in sensor technology, miniaturization, and data analytics, the market for health care wearables has witnessed exponential growth in recent years. The global market for health care wearables was valued at US $33.85 billion in 2023 and is projected to reach US $250 billion by 2030 [13], driven by increasing consumer demand for continuous health monitoring and the growing adoption of telehealth services.

Wearable health care devices have been found to be effective in gathering reliable health information for various conditions including cardiovascular diseases [14-17], chronic respiratory conditions like asthma, and chronic obstructive pulmonary disease [18-20], Parkinson disease [21-23], and mental health conditions such as stress, anxiety, depression, and behavioral changes [24-27]. Despite such promising benefits, adoption rates of wearable health care devices have varied across different demographics and health conditions [28-30]. While some individuals enthusiastically embrace these technologies for health monitoring purposes, others remain skeptical or face barriers such as cost, usability issues, and concerns regarding data privacy and security [31-33]. For instance, studies have indicated that older adults [34,35], individuals with lower income [36,37], and those with limited digital literacy [38-40] may experience greater difficulties in accessing and using wearable technology. In addition, questions have also been raised about the accuracy and reliability of data captured by wearable devices [14,41,42]. Therefore, to fully realize the potential benefits offered by wearable health care devices, effective sharing of wearable health data from patients to health care providers is critical.

**Figure 1:**
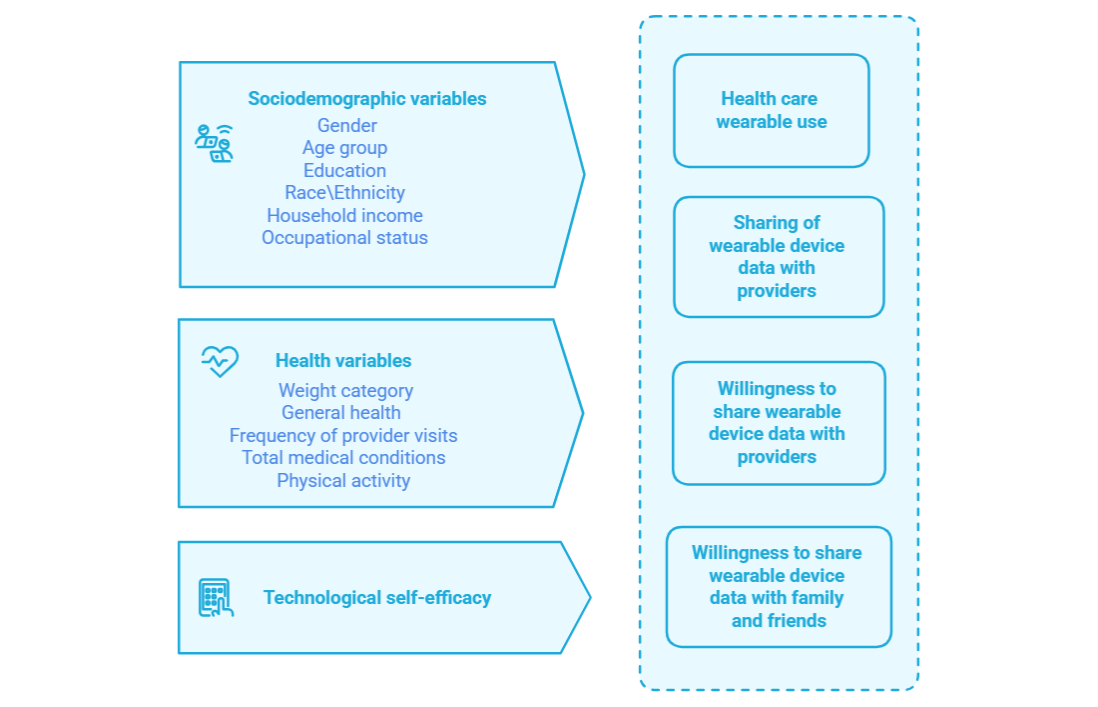
Research model.

Many studies have examined individuals’ behavioral intentions to use wearable devices [43-46] and their willingness to share health data generated by wearables with health care providers [47,48]. Other studies have documented the willingness of individuals to share the data generated from their wearable health devices for research purposes [49-51]. However, despite documented intentions, there remains a notable gap in understanding the actual, actionable health data-sharing behaviors of users of health care wearable devices. This study aims to address this gap by investigating both self-reported willingness and actual behavior in sharing health data generated by wearable devices. We present findings based on a national survey conducted among US adults to ascertain the current landscape of health care wearable usage. We build upon and extend our earlier work [30] that examined wearable usage in the prepandemic period (2019) by using a recent dataset from 2022 to provide insights into the current use of wearable devices, with a particular focus on wearable data-sharing practices. In addition, we explore potential disparities in wearable use across socioeconomic demographics, health conditions, levels of technological self-efficacy, and engagement in physical activities. Furthermore, we examine whether these factors are associated with variations in individuals’ willingness to share health data with health care providers and family or friends. Importantly, we assess the actual data-sharing behavior to identify predictors associated with the willingness-action gap and compare our findings with similar studies to highlight both similarities and differences (Figure 1). 

This study leverages the most recent, nationally representative dataset of US adults, gathered in the later phases of COVID-19 pandemic (2022), a period marked by heightened health awareness and evolving health behaviors [52]. As we transition into the postpandemic era, understanding the use and data-sharing behaviors associated with wearable devices has become increasingly critical. The data collected during this period could offer valuable insights into shifts in health behaviors and highlights the role of wearable technology in managing and improving health engagement.

This study aims to answer the following questions:

1. How has wearable device usage among US adults changed since 2019, and what factors are associated with wearable use in the postpandemic era?
2. What are the key sociodemographic, health, and technological factors that influence US adults’ willingness to share health data from wearable devices with health care providers, and with their family and friends?
3. How do actual data-sharing behaviors with health care providers compare with individuals’ self-reported willingness to share wearable health data, and what are the key predictors of the actual data-sharing behavior?

## Methods

### Sample and Data Collection

Data for this study were sourced from the sixth cycle of the Health Information National Trends Survey (HINTS 6), conducted by the National Cancer Institute from March through November 2022. HINTS 6 is a nationally representative, probability-based cross-sectional survey. In this cycle, adult, civilian, noninstitutionalized individuals were invited to participate through self-administered questionnaires, with respondents completing the survey either on paper or online. The survey used a nationally representative sampling strategy, targeting areas with high concentrations of minorities and those with lower minority concentrations, in addition to stratification by rural and urban geographic areas.

The survey was designed to collect data in 2 modes, giving respondents the option to complete the questionnaire through paper or through the web, with certain incentives offered for web participation. The sampling method continued to use a 2-stage design, initially selecting a stratified sample of addresses, followed by the selection of 1 adult per household from these addresses. This methodology ensures a broad representation of the US population, though the reliance on self-reported data may introduce certain biases in reporting and recall. To include respondents in the analytic sample, we implemented a 2-step filtering process: (1) respondents must have reported having a smart device, such as a smartphone or tablet, or multiple smart devices, and (2) they must have provided a response to the question regarding their use of a wearable device for tracking health or activity in the past 12 months. Specifically, only those smart device owners who answered the question, “In the last 12 months, have you used an electronic wearable device to monitor or track health or activity? For example, a Fitbit, Apple Watch, or Garmin Vivofit,” were included in the dataset for analysis. This approach resulted in 5591 completed survey responses, ensuring consistency with our research goals pertaining to current usage patterns of wearable health care devices and also into the data-sharing behaviors with health care providers and personal networks.

### Variables and Measures

#### Outcome Variables

The primary focus of this study was on the use of wearable health care devices, assessed through a binary (yes or no) question asking respondents if they had used such an electronic wearable device to monitor or track their health or activity in the past 12 months. In addition, we also explored the responses about the frequency of wearable health care device use in the past month (everyday, almost every day, 1-2 times a week, <1 time a week, never used). In addition, the study explored respondents’ willingness to share data from their wearables with health care providers and with family and friends, also assessed as yes or no responses. To understand actual data-sharing behavior, we examined a survey question about whether respondents had shared health information from a wearable device with a health professional in the last 12 months.

#### Independent Variables

The study incorporated data from the survey questionnaire to capture respondents’ self-reported characteristics, which included sociodemographic factors (such as age, gender, race/ethnicity, education, occupational status, and annual household income), health-related aspects (general health status, the total number of medical conditions, frequency of health care provider visits, and weight category), and technology-related factors (technology self-efficacy). General health was evaluated on a Likert scale from 1 (poor) to 5 (excellent). To quantify the total number of medical conditions, we counted 5 self-reported conditions such as diabetes or high blood sugar, hypertension, heart conditions (eg, heart attack, angina, or congestive heart failure), chronic lung diseases (such as asthma, emphysema, or chronic bronchitis), and mental health issues like depression or anxiety disorders. The values for total medical conditions ranged from 0 to 5. The frequency of health care provider visits was measured on a scale based on the number of visits in the past year, with values ranging from 0 (none) to 6 (10 times or more). Weight categories were determined based on BMI values: a BMI under 18.5 indicates underweight, and values between 18.5 and 25 indicates a normal healthy status, 25 to 29.9 is considered overweight, and a BMI of 30 or above is categorized as obese.

Previous research has demonstrated that technological self-efficacy, or comfort with technology, significantly influences the use of wearables [35,53,54]. Building on these findings, we measured respondents’ technological self-efficacy based on their engagement in 4 internet-based health activities over the past year: searching for health information, electronic communication with health providers, accessing medical test results online, and scheduling appointments online. Each activity was scored as yes (1) or no (0), with total scores ranging from 0 to 4, indicating increasing comfort with technology.

We also explored the relationship between physical activity exercise routines and wearable device usage. Previous research suggests that individuals who are regularly motivated to exercise and engage in physical activities are more inclined to adopt and use wearable devices [55,56]. To assess the physical activity levels of respondents, we examined a question asking how many days per week they engage in any physical activity or exercise of at least moderate intensity (0-7 days a week). Examples provided included brisk walking, bicycling at a regular pace, and swimming at a regular pace.

### Statistical Analysis

All the statistical analyses were performed using STATA software (version 18; StataCorp). We began with a descriptive analysis of our data sample. To explore the associations between the use of wearable health care devices and various sociodemographic predictors, we generated crosstab tables and evaluated them using the Wald chi-square test. This approach was repeated to analyze the willingness to share wearable health data with providers, family, and friends, as well as the actual sharing of such data with health care providers.

Given that our primary variables of interest were all binary, and our predictor variables included both categorical and continuous types, logistic regression analysis was used (Figure 1). We ran 4 survey logistic regression models (using svy: logistic command in STATA) with the following outcome variables: (1) use of wearable health care device (0/1), (2) willingness to share health care wearable data with providers (0/1), (3) willingness to share health care wearable data with family and friends (0/1), and (4) actual sharing of wearable data with providers (0/1). The predictor variables were the sociodemographic factors (gender, age group, education level, race/ethnicity, annual household income, and occupation status), health related variables (weight category based on BMI, self-reported general health, frequency of provider visits, total medical conditions, and extent of physical activity) and technology self-efficacy. We calculated adjusted odds ratios (ORs) and CIs for these predictors. To account for the complex survey design, we applied weights with jackknife replications in our statistical analyses, using *svy:* commands in STATA. Since the svy: logistic model in STATA does not assess multicollinearity, we initially ran standard logistic regressions and examined variance inflation factor values with a cutoff value of 4, before performing svy: logistic regression assessments.

### Ethical Considerations

HINTS data are publicly available and collected as deidentified information from participants, specifically designed for research purposes [57]. The HINTS 6 survey was classified as “exempt research” under 45 CFR 46.104 and approved by the Westat institutional review board (IRB) on May 10, 2021 (Project #6632.03.51), with an amendment approved on November 24, 2021 (Amendment ID #3597). It also received a “Not Human Subjects Research” determination from the National Institutes of Health (NIH) Office of IRB Operations on August 16, 2021 (iRIS reference #562715). Therefore, no additional approvals were required from authors’ institutions. Further details on the survey instrument, and the methodology and data handling for HINTS 6 can be explored through the National Cancer Institute’s official resources [58,59].

## Results

### Overview

Table 1 describes the characteristics of survey respondents. A total of 5591 respondents had indicated having a smart device that is needed for storing and sharing data from a health care wearable device, in addition to responding to the question about use of a wearable device. A total of 3161 out of 5591 (60.45%) were female, 3405 out of 5591 (61.55%) were aged 50 years or older, 2600 out of 5591 (49.67%) had a college degree or higher, and 2479 out of 5591 (44.34%) were employed full-time. Among the respondents, 2925 out of 5591 (57.97%) identified their race\ethnicity as White, 785 out of 5591 (15.56%) as Black, 893 out of 5591 (17.7%) as Hispanic, and 271 out of 5591 (5.37%) as Asian. Furthermore, 2100 out of 5591 (42.24%) indicated their annual household incomes to be US $75,000 or more.

**Table 1:** Socioeconomic demographic characteristics of survey respondents.

Characteristics		Survey respondents, n (%)
**Gender (n=5229)**		
	Male	2068 (39.55)
	Female	3161 (60.45)
**Age group (years; n=5532)**		
	18-34	927 (16.76)
	35-49	1200 (21.69)
	50-64	1639 (29.63)
	65-74	1168 (21.11)
	75+	598 (10.81)
**Education (n=5235)**		
	Less than high school	268 (5.12)
	High school	849 (16.22)
	Some college	1518 (29)
	College graduate or more	2600 (49.67)
**Race\Ethnicity (n=5046)**		
	Non-Hispanic White	2925 (57.97)
	Non-Hispanic African American	785 (15.56)
	Hispanic	893 (17.7)
	Non-Hispanic Asian	271 (5.37)
	Others	172 (3.41)
**Household income (US $; n=4972)**		
	< 20,000	719 (14.46)
	20,000 to <35,000	613 (12.33)
	35,000 to <50,000	666 (13.4)
	50,000 to <75,000	874 (17.58)
	> 75,000	2100 (42.24)
**Occupational status (n=5591)**		
	Employed full-time	2479 (44.34)
	Employed part-time	366 (6.55)
	Not employed or retired	2768 (49.11)

### Health Care Wearable Use and Frequency of Use

**Figure 2:**
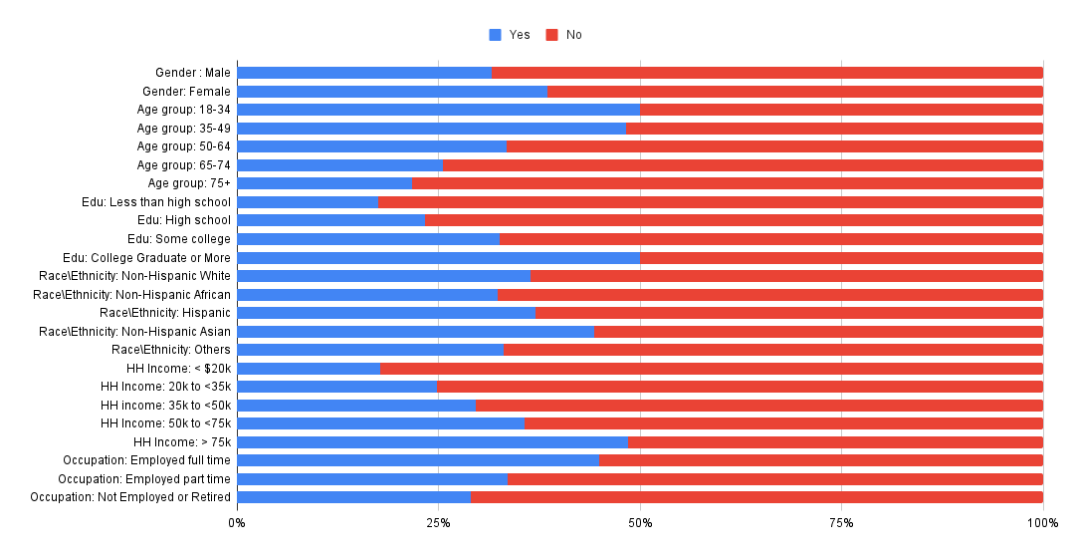
Health care wearable use across different demographic segments.

Out of 5591 respondents, 2033 (36.36%) reported using a health care wearable device in the past 12 months (Table 2). In the exploratory analysis, we observed statistically significant differences in wearable use (Table 3) across age groups (*χ*^2^_4_=261.7, *P*<.01), gender (*χ*^2^_1_=25.7, *P*<.01), education (*χ*^2^_3_=170.6, *P*<.01), occupation status (*χ*^2^_2_=143.1, *P*<.01), race\ethnicity (*χ*^2^_4_=13.7, *P*<.01) and income levels (*χ*^2^_4_=290.1, *P*<.01; Figure 2). When asked about health care wearable use in the past month, 880 out of 5591 (43.48%) reported daily use, 557 out of 5591 (27.52%) used them almost every day, 217 out of 5591 (10.72%) used them 1-2 times per week, and 156 out of 5591 (7.71%) used them less than once a week. Notably, 214/5591 (10.57%) indicated they had not used their wearables at all in the past month.

**Table 2:** Usage, willingness, and actual sharing of wearable health data.

Variable	Response	
	Yes, n (%)	No, n (%)
Health care wearable device use (n=5591)	2033 (36.4)	3558 (63.6)
Health information sharing from wearable device (n=2020)	535 (26.5)	1485 (73.5)
Willingness to share wearable health data with provider (n=2020)	1584 (78.4)	436 (21.6)
Willingness to share wearable health data with family and friends (n=2014)	1266 (62.9)	748 (37.1)

**Table 3:** Variations in wearable use based on demographics.

Respondent demographics		Use of wearable health care device in the past 12 months (n=5591)					
		Yes, n (%)	No, n (%)	Chi-square (*df*)		*P* values	
**Gender**					25.7 (1)		<.01
	Male	654 (12.51)	1414 (27.04)				
	Female	1217 (23.27)	1944 (37.18)				
**Age group (years)**					267.7 (4)		<.01
	18-34	463 (8.37)	463 (8.37)				
	35-49	579 (10.47)	621 (11.23)				
	50-64	549 (9.93)	1090 (19.71)				
	65-74	298 (5.39)	870 (15.73)				
	75+	130 (2.35)	468 (8.46)				
**Education**					170.6 (3)		<.01
	Less than high school	47 (0.9)	221 (4.22)				
	High school	193 (3.78)	651 (12.44)				
	Some college	494 (9.44)	1024 (19.56)				
	College graduate or more	1131 (21.6)	1469 (28.06)				
**Race\ethnicity**					13.7 (4)		<.01
	Non-Hispanic White	1065 (21.11)	1860 (36.86)				
	Non-Hispanic African American	254 (5.03)	531 (10.52)				
	Hispanic	331 (6.56)	562 (11.14)				
	Non-Hispanic Asian	120 (2.38)	151 (2.99)				
	Others	57 (1.13)	115 (2.28)				
**Household income (US $)**					290.1 (4)		<.01
	< 20k	128 (2.57)	591 (11.89)				
	20k to <35k	152 (3.06)	461 (9.27)				
	35k to <50k	197 (3.96)	469 (9.43)				
	50k to <75k	312 (6.28)	562 (11.30)				
	> 75k	1019 (20.49)	1081 (21.74)				
**Occupation status**					143.2 (2)		<.01
	Employed full time	1113 (19.91)	1366 (24.43)				
	Employed part time	123 (2.2)	243 (4.35)				
	Not employed or retired	797 (14.26)	1949 (34.86)				

### Wearable Data Sharing: Willingness and Sharing Behavior

Out of 2020 wearable users, 1584 (78.4%) expressed willingness to share data from health care wearables with their providers, and 1266 out of 2014 (62.95%) users indicated willingness to share health data with family or friends. Despite this willingness, only 535 out of 2020 (26.5%) users actually shared data from wearable devices with providers within the past 12 months (Table 2). This highlights a significant gap between the intention to share health data and actual sharing behavior. Table 4 reveals demographic disparities in health data sharing behaviors based on exploratory chi-square tests: gender (*χ*^2^_1_=4.08, *P*=.04), age groups (*χ*^2^_4_=50.45, *P*<.01), race/ethnicity (*χ*^2^_4_=12.79, *P*<.01), and occupational status (*χ*^2^_2_=18.56, *P*<.01). Differences in the willingness to share wearable data with health providers were significant only by race/ethnicity (*χ*^2^_4_=10.35, *P*<.03). Similarly, the willingness to share data with family and friends showed significant variation across different age groups (*χ*^2^_4_=20.44, *P*<.01) and occupational status (*χ*^2^_2_=5.64, *P*<.05).

**Table 4:** Variations in data sharing and willingness to share wearable data based on demographics.

Respondent demographics		Willingness to share wearable data with health care provider (n=2023)							Willingness to share wearable data with family or friends (n=2014)									Actual sharing of health data with health professional in the past 12 months (n=2020)						
		Yes, n (%)	No, n (%)	Chi-square (*df*)		*P* value		Yes, n (%)		No, n (%)		Chi-square (*df*)		*P* value		Yes, n (%)			No, n (%)		Chi-square (*df*)		*P* value	
**Gender**					1.3 (1)		<.25						0.2 (1)		<.68							4.1 (1)		<.05
	Male	520 (27.96)	129 (6.94)					403 (21.73)		244 (13.15)						190 (10.22)			463 (24.89)					
	Female	943 (50.7)	268 (14.41)					764 (41.19)		444 (23.94)						299 (16.08)			908 (48.82)					
**Age group (years)**					4.9 (4)		<.29						20.4 (4)		<.01							50.4 (4)		<.01
	18-34	355 (17.69)	105 (5.23)					317 (15.83)		143 (7.14)						92 (4.59)			367 (18.3)					
	35-49	445 (22.17)	132 (6.58)					384 (19.18)		193 (9.64)						122 (6.08)			454 (22.63)					
	50-64	426 (21.23)	119 (5.93)					311 (15.53)		233 (11.64)						156 (7.78)			392 (19.54)					
	65-74	244 (12.16)	52 (2.59)					173 (8.64)		121 (6.04)						103 (5.13)			194 (9.67)					
	75+	106 (5.28)	23 (1.15)					77 (3.85)		50 (2.5)						56 (2.79)			70 (3.49)					
**Education**					4.8 (3)		<.19						1.9 (3)		<.58							4.6 (3)		<.21
	Less than high school	34 (1.83)	13 (0.7)					25 (1.35)		20 (1.08)						9 (0.48)			37 (1.99)					
	High school	145 (7.8)	50 (2.69)					122 (6.58)		72 (3.88)						43 (2.31)			152 (8.18)					
	Some college	382 (20.55)	110 (5.92)					300 (16.18)		190 (10.25)						142 (7.64)			350 (18.83)					
	College Graduate or More	900 (48.41)	225 (12.1)					717 (38.67)		408 (22.01)						295 (15.87)			831 (44.7)					
**Race\ethnicity**					10.3 (4)		<.03						6.9 (4)		<.14							12.8 (4)		<.01
	Non-Hispanic White	855 (47.06)	206 (11.34)					682 (37.6)		378 (20.84)						279 (15.35)			781 (42.98)					
	Non-Hispanic African American	193 (10.62)	58 (3.19)					140 (7.72)		110 (6.06)						81 (4.46)			171 (9.41)					
	Hispanic	259 (14.25)	71 (3.91)					205 (11.3)		124 (6.84)						69 (3.8)			260 (14.31)					
	Non-Hispanic Asian	81 (4.46)	37 (2.04)					73 (4.02)		45 (2.48)						26 (1.43)			93 (5.12)					
	Others	47 (2.59)	10 (0.55)					39 (2.15)		18 (0.99)						20 (1.1)			37 (2.04)					
**Household income (US $)**					0.9 (4)		<.91						6.9 (4)		<.13							6.9 (4)		<.14
	<20,000	98 (5.45)	30 (1.67)					67 (3.74)		59 (3.29)						42 (2.34)			82 (4.56)					
	20,000 to <35,000	118 (6.57)	34 (1.89)					93 (5.19)		59 (3.29)						42 (2.34)			110 (6.12)					
	35,000 to <50,000	154 (8.57)	41 (2.28)					129 (7.19)		65 (3.63)						57 (3.17)			139 (7.73)					
	50,000 to <75,000	249 (13.86)	61 (3.39)					200 (11.15)		109 (6.08)						73 (4.06)			236 (13.13)					
	> 75,000	799 (44.46)	213 (11.85)					643 (35.86)		369 (20.58)						252 (14.02)			765 (42.55)					
**Occupation status**					0.4 (2)		<.80						5.6 (2)		<.05							18.6 (2)		<.1
	Employed full time	862 (42.67)	245 (12.13)					712 (35.35)		394 (19.56)						258 (12.77)			852 (42.18)					
	Employed part time	97 (4.8)	26 (1.29)					84 (4.17)		39 (1.94)						37 (1.34)			96 (4.75)					
	Not employed or retired	625 (30.94)	165 (8.17)					470 (23.34)		315 (15.64)						250 (12.38)			537 (26.58)					

### Predictors of Health Care Wearable Use

Table 5 presents results from a logistic regression analysis examining predictors of health care wearable use. Women, compared with men, are more likely to use health care wearables (OR 1.49, 95% CI 1.17-1.9, *P*<.01). The likelihood of using health care wearables declines with age; compared with the 18-34 years age group, the 50-64 years (OR 0.49, 95% CI 0.36-0.67, *P*<.01), 65-74 years (OR 0.37, 95% CI 0.26-0.54, *P*<.01), and over 75 years (OR 0.35, 95% CI 0.20-0.61, *P*<.01) age groups showed significantly lower odds. Higher annual household incomes are also associated with increased likelihood of wearable use, notably in the US $50,000 to US $75,000 range (OR 2.65, 95% CI 1.42-4.93, *P*<.01) and above US $75,000 (OR 3.21, 95% CI 1.71-5.97, *P*<.01). Weight categories, as assessed by BMI significantly influenced wearable use. Overweight individuals (BMI 25-29.9) were more likely to use wearables compared with those with normal weight (BMI 18.5-24.9; OR 1.36, 95% CI 0.98-1.89, *P*<.05). Conversely, underweight individuals (BMI<18.5) were less likely to use wearables (OR 0.31, 95% CI 0.09-1.04, *P*<.05). Obese individuals (BMI≥30) also showed higher odds of use (OR 1.33, 95% CI 0.93-1.90, *P*<.10), though this was only significant at the 10% level.

Among the health-related variables, the frequency of provider visits was significantly associated with an increase in wearable use (OR 1.08, 95% CI 1.02-1.15, *P*<.01), whereas neither self-rated general health nor the number of medical conditions were significant predictors. In addition, our analysis indicated that individuals who exercised more frequently were more likely to use wearables (OR 1.15, 95% CI 1.11-1.20, *P*<.01).

As reported in Table 5, our findings indicated a strong association between technological self-efficacy and wearable use (OR 1.29, 95% CI 1.15-1.44, *P*<.01).

**Table 5:** Factors associated with health care wearable use: results of logistic regression.

Predictors and subcategory		Odds ratio (95% CI)	*P* value
**Gender (reference category: male)**			
	Female	1.49 (1.17-1.90)	.01
**Age group (years; reference category: 18-34 years)**			
	35-49	1.09 (0.79-1.51)	.57
	50-64	0.49 (0.36-0.67)	<.001
	65-74	0.37 (0.26-0.54)	<.001
	75+	0.35 (0.2-0.61)	<.001
**Education (reference category: college graduate or higher)**			
	Less than high school	0.78 (0.38-1.62)	.50
	High school	0.76 (0.54-1.07)	.11
	Some college	0.89 (0.66-1.19)	.43
**Race/ethnicity (reference category: non-Hispanic African American)**			
	Non-Hispanic White	1.19 (0.87-1.65)	.25
	Hispanic	1.38 (0.99-1.91)	.06
	Non-Hispanic Asian	1.39 (0.67-2.85)	.36
	Others	1.15 (0.59-2.22)	.67
**Household income (US $; reference category: less than US $20,000)**			
	20,000 to <35,000	1.61 (0.82-3.13)	.15
	35,000 to <50,000	1.58 (0.84-2.96)	.15
	50,000 to <75,000	2.65 (1.42-4.93)	.01
	75,000 or more	3.21 (1.71-5.97)	.01
**Occupational status (reference category: not employed or retired)**			
	Employed full-time	1.02 (0.79-1.31)	.85
	Employed part-time	0.89 (0.51-1.54)	.68
**Weight category (reference category: normal weight)**			
	Underweight	0.31 (0.09-0.84)	.05
	Overweight	1.36 (1.08-1.89)	.05
	Obesity	1.33 (0.93-1.9)	.11
**Health**			
	General health	1.02 (0.87-1.21)	.77
	Frequency of provider visits	1.08 (1.02-1.15)	.01
	Total medical conditions	0.96 (0.83-1.11)	.58
	Physical activity (times exercise)	1.15 (1.11-1.2)	.01
**Technology self-efficacy**			
	Electronic usage	1.29 (1.15-1.44)	.01

### Predictors of Health Data Sharing and Willingness to Share Wearable Data

A series of 3 logistic regressions were used to explore the relationships between various predictors and both the behavior of sharing health data and the willingness to share data from wearable health devices with providers, family, and friends. These findings are detailed in Table 6.

While women were more likely to use health care wearable devices compared with men, they were less likely to share the data from these devices with providers (OR 0.66, 95% CI 0.44-0.99, *P*<.05). No significant associations were found between gender and the willingness to share wearable data with either providers or family and friends.

**Table 6:** Key predictors of wearable data sharing behavior and willingness to share wearable data: logistic regression results.

Predictors and subcategory		Wearable health data sharing with provider		Willingness to share wearable data with provider		Willingness to share wearable data with family and friends	
		Odds ratio (95% CI)	*P* values	Odds ratio (95% CI)	*P* values	Odds ratio (95% CI)	*P* values
**Gender (Ref: male)**							
	Female	0.66 (0.44-0.99)	.04	0.80 (0.54-1.2)	.27	1.13 (0.83-1.54)	.42
**Age group (years; ref: 18-34 years)**							
	35-49	1.08 (0.61-1.92)	.80	1.14 (0.66-1.99)	.63	0.68 (0.4-1.18)	.17
	50-64	1.47 (0.82-2.64)	.19	0.94 (0.48-1.86)	.85	0.39 (0.22-0.71)	.01
	65-74	1.64 (0.74-3.63)	.22	1.26 (0.48-3.28)	.63	0.35 (0.21-0.59)	.01
	75+	4.14 (1.1-14.6)	.03	2.52 (0.74-8.64)	.14	0.46 (0.23-0.93)	.03
**Education (ref: college graduate or higher)**							
	Less than High School	0.95 (0.26-3.45)	.94	1.03 (0.33-3.29)	.95	0.72 (0.1-4.98)	.73
	High School	0.78 (0.36-1.71)	.53	0.66 (0.3-1.46)	.3	1.11 (0.6-2.06)	.73
	Some College	1.36 (0.91-2.02)	.13	1.15 (0.64-2.06)	.64	1.14 (0.8-1.65)	.46
**Race/ethnicity (ref: Non-Hispanic African American)**							
	Non-Hispanic White	0.53 (0.25-1.14)	.10	1.74 (1.08-3.09)	.05	1.81 (1.04-3.16)	.04
	Hispanic	0.44 (0.2-0.97)	.05	1.92 (1.02-3.62)	.04	1.34 (0.74-2.42)	.32
	Non-Hispanic Asian	0.41 (0.13-1.23)	.11	0.87 (0.39-1.92)	.72	1.02 (0.45-2.31)	.95
	Others	0.66 (0.21-2.03)	.46	2.57 (0.6-11.09)	.20	2 (0.8-5.02)	.13
**Household income (US $; ref: Less than US $20,000)**							
	20,000 to <35,000	0.34 (0.08-1.35)	.12	0.96 (0.29-3.12)	.94	0.98 (0.39-2.46)	.95
	35,000 to <50,000	0.45 (0.12-1.65)	.22	0.55 (0.2-1.53)	.25	1.33 (0.51-3.42)	.55
	50,000 to <75,000	0.28 (0.08-0.94)	.04	0.93 (0.32-2.69)	.89	1.1 (0.41-2.91)	.84
	75,000 or more	0.31 (0.09-0.99)	.05	0.60 (0.22-1.67)	.32	1.02 (0.45-2.27)	.96
**Occupational status**							
	Employed Full time	0.87 (0.49-1.55)	.63	0.83 (0.47-1.45)	.51	0.97 (0.63-1.51)	.90
	Employed Part time	0.88 (0.32-2.41)	.80	1.38 (0.53-3.61)	.50	1.78 (0.87-3.61)	.11
**Weight category**							
	Underweight	4.29 (0.66-27.56)	.12	1.92 (0.51-7.30)	.33	0.82 (0.23-2.94)	.76
	Overweight	1.34 (0.85-2.11)	.20	1.34 (0.79-2.26)	.28	0.97 (0.61-1.55)	.90
	Obesity	1.42 (0.89-2.27)	.14	1.40 (0.85-2.3)	.19	0.96 (0.61-1.52)	.86
**Health**							
	General Health	1.16 (0.84-1.60)	.35	1.10 (0.85-1.42)	.46	1.21 (0.97-1.52)	.09
	Frequency of Provider Visits	1.23 (1.08-1.39)	.01	1.29 (1.08-1.43)	.04	1.10 (0.98-1.23)	.15
	Total Medical Conditions	1.35 (1.05-1.73)	.01	1.06 (0.87-1.29)	.53	1.06 (0.84-1.32)	.54
	Physical Activity (Times Exercise)	1.09 (0.98-1.21)	.10	0.99 (0.91-1.07)	.81	1.08 (1.07-1.17)	.05
**Technology self-efficacy**							
	Electronic Usage	1.26 (0.92-1.71)	.14	1.13 (0.94-1.36)	.17	0.94 (0.8-1.11)	.46

Older adults aged 75 years and above showed higher odds of sharing wearable device data with providers (OR 4.14, 95% CI 1.18-14.6, *P*<.01) compared with the younger (18-34 years) age group. Although no significant differences were noted among age groups in their willingness to share wearable data with providers, a negative association was observed in their willingness to share with family and friends. Specifically, individuals aged 50-64 years (OR 0.39, 95% CI 0.22-0.71, *P*<.01), 65-74 years (OR 0.35, 95% CI 0.23-0.93, *P*<.01), and older than 75 years (OR 0.46, 95% CI 0.23-0.93, *P*<.05) were less likely to share health data from wearable devices with family or friends.

Compared with non-Hispanic Black respondents, Hispanic respondents (OR 1.92, 95% CI 1.02-3.62, *P*<.05) and non-Hispanic White respondents (OR 1.74, 95% CI 1.08-3.09, *P*<.05) exhibited higher odds of willingness to share health data from wearables with providers. Furthermore, non-Hispanic White respondents showed a greater tendency to share wearable data with family and friends (OR 1.81, 95% CI 1.04-3.16, *P*<.05). However, in comparison with non-Hispanic Black respondents, actual sharing behavior was lower for Hispanic respondents (OR 0.44, 95% CI 0.20-0.97, *P*<.05). Altogether, these results indicate racial disparities in the actual sharing of health data despite a higher willingness.

No significant differences were observed in the willingness to share health data with providers, as well as family and friends, based on annual household income, yet the likelihood of sharing health data with providers decreased with higher income levels. Those earning between US $50,000 and US $75,000 (OR 0.28, 95% CI 0.08-0.94, *P*<.05), and those with incomes above US $75,000 (OR 0.31, 95% CI 0.09-0.99, *P*<.05) showed lesser odds of sharing.

Frequency of provider visits emerged as a significant predictor for both willingness to share and actual sharing behavior with providers. Those who visited their providers more frequently were more willing to share data from wearable devices (OR 1.29, 95% CI 1.08-1.43, *P*<.05) and more actively engaged in sharing this data (OR 1.23, 95% CI 1.08-1.39, *P*<.01). Individuals with more medical conditions also showed higher odds of sharing data with providers (OR 1.35, 95% CI 1.05-1.73, *P*<.01). A weak association was observed between self-reported general health and the willingness to share data with family and friends (OR 1.21, 95% CI 0.97-1.52, *P*<0.1). In addition, individuals engaging in more exercise and physical activities were found more likely to share wearable health care data with family and friends (OR 1.08, 95% CI 1-1.17, *P*<.05).

## Discussion

### Principal Findings

Wearable health care devices such as smartphones and activity trackers offer continuous and real-time monitoring of an individual’s health metrics. These tools are becoming increasingly popular as they not only empower users to take proactive control over their health but also provide health care providers with invaluable data for better patient care. Despite their potential, significant gaps remain in understanding the predictors that influence wearable device use, and data-sharing behaviors associated with these devices. This study aimed to address these gaps by identifying key factors that influence both the use of wearable devices, the willingness to share the data they collect and actual data-sharing behavior. We observed an increase in wearable device usage from 28%-30% in 2019 to 36.36% in 2022, reflecting a broader trend of heightened health awareness, particularly following the COVID-19 pandemic. In addition, despite a high willingness to share wearable data with health care providers and family or friends, there is a significant gap between willingness and actual sharing behaviors, with only a quarter of users actively sharing data with providers.

Our results highlight a significant uptick in the adoption and use of health care wearable devices, from 28%-30% in 2019 [30,60] and 21% according to a 2020 Pew Research Study [61], to 36.36% in 2022. Our findings from HINTS 6 dataset, collected during a period of accelerating vaccinations and evolving public health measures, reflect how the COVID-19 pandemic has increased the adoption of wearable devices and shifted consumer attitudes toward self-health monitoring. The rise in wearable usage in 2022 signifies a growing health consciousness and commitment to self-health monitoring, suggesting that wearable technology will be crucial in fostering ongoing health engagement and preventive care in the postpandemic landscape [62,63].

Our analysis found that 78.4% of US adults were willing to share data from wearable devices with providers, and 62.95% were willing to share with family and friends. However, only 26.5% actually shared data with providers. This willingness-action gap may stem from factors such as privacy concerns and technological challenges. Notably, there is a decrease in willingness compared with previous years; studies from 2019 reported that 81.85% of respondents were willing to share wearable data with providers, and 69.51% with family or friends [47]. Other studies have reported even higher levels of willingness [30,48], particularly among subgroups such as cardiovascular patients and older individuals [35,64]. This decline in willingness suggests that while interest in wearable technology remains high, barriers to data sharing may have increased, hindering the translation of willingness into actual sharing behavior with providers.

Our analysis also reveals that wearable use is not uniform across demographic segments. Notably, women are more likely than men to use these devices. This trend is partly attributed to women’s greater health consciousness and proactive engagement in health monitoring and management activities [65,66]. Previous research has also found that women with one or more chronic conditions are more likely to use wearable devices [67]. However, we also find that women are less likely to share data from wearable devices with health providers. This reluctance, relatively more in women, may stem from privacy concerns, fear of data misuse, and lack of trust in how their health information will be handled [68,69]. It is essential to develop targeted education and intervention programs for women that emphasize benefits of data sharing for personalized health care and improved medical outcomes. In addition, ensuring robust data security measures and transparent communication about how data will be used can help build trust and encourage more individuals to share their health information from wearable devices.

We also find that wearable usage decreases with age, with individuals over 75 showing the least likelihood of using wearables. This finding is consistent with previous studies [35]. This decline could be attributable to varying levels of technological comfort or perceived use and usability of the devices among the older adults [34,70]. Furthermore, older adults could find the devices to be too complex to use and integrate into their daily lives [71]. However, we also found older adults to be less willing to share wearable device data with family and friends but were actually more engaged in sharing this data with their providers. This behavior suggests that older adults may prioritize sharing health information with professionals for medical oversight and care management, highlighting a trust in professional health care settings over personal networks.

Racial and ethnic disparities also manifest in wearable device use, with Hispanic individuals showing a greater propensity to share data from these devices compared with non-Hispanic Black individuals. This could reflect cultural differences in health management practices or disparities in access to technology. Our findings are consistent with previous studies that have documented increased interest in wearable devices among Hispanic communities, especially after the COVID-19 pandemic [72]. Challenges in obtaining health resources and general distrust in health care infrastructure and systems have also motivated Hispanic individuals to look at wearable devices to better monitor their health care. Hispanic individuals have also been engaging more with electronic tools to acquire health information using online resources and for communicating with providers [73]. While non-Hispanic White and Hispanic individuals showed a higher willingness to share data with providers, their actual sharing rates did not align with these intentions, indicating possible systemic or personal barriers that prevent translating willingness into action.

Our findings reveal that economic factors significantly influence wearable device usage, with individuals having higher annual household incomes being more likely to use these technologies, likely due to greater access to and affordability of emerging health technologies. Interestingly, despite their higher use rates, these individuals are less inclined to share wearable data with health care providers, possibly due to heightened privacy concerns [74,75] or a greater sense of autonomy in managing their health data independently.

Our analysis extends into health-related variables and conditions influencing wearable adoption. Frequent visits to health care providers and the presence of multiple health conditions are associated with higher likelihood of wearable usage and sharing of wearable data with providers, suggesting that individuals more engaged with their health are more receptive to using technology to manage their health. Our findings complement previous studies that have documented wearable use among patients with cardiovascular conditions [76], and diabetes [77,78].

Furthermore, our findings suggest that individuals who regularly engage in physical activities are not only more likely to use wearables but are also more willing to share the data generated, especially with family and friends potentially to monitor their fitness progress or health status more effectively. Many users share their physical activities with a group of friends using fitness and health apps, which helps to boost motivation and receive positive reinforcement [79,80]. These social features and gamification in wearable health apps allow users to join challenges, share achievements, and provide mutual support, significantly enhancing adherence to fitness goals and creating a sense of community [81].

### Implications

The findings from this study on wearable health care devices suggest several implications for health care providers, policy makers, regulators, and industry stakeholders. Wearable devices can provide health data relevant to users’ specific health needs and concerns, yet access to these benefits is not equally distributed among all users. Those with greater digital literacy and socioeconomic resources tend to have better access to the health monitoring and predictive capabilities of wearable technologies.

Health care providers can enhance wearable data sharing by addressing patient concerns, particularly among women and older adults, through targeted education and communication that underscores the benefits of sharing health wearable data. There is also a need for simplified training programs to increase wearable device use among older adults. Policy makers and regulators should focus on improving accessibility and affordability for lower-income individuals, ensuring robust data protection standards, and promoting interoperability between wearable devices and health systems to build trust and use. For racial and ethnic minorities, culturally tailored programs that address disparities in technology access and use are crucial. In addition, health insurers and technology companies could offer incentives for wearable data sharing and develop features that enhance user engagement through social connectivity and gamification, fostering a community-focused approach to health management. Collectively, these efforts can lead to better integration of wearable technologies in health care, providing valuable insights for preventive health measures and personalized care, thereby maximizing the potential of digital health tools in improving public health outcomes.

### Limitations

This study has several limitations. Primarily, it relies on self-reported information by survey respondents, which can be subject to recall bias and social desirability bias. Respondents may not accurately remember past behaviors or may portray themselves in a more favorable light. For some variables, which may introduce inherent biases. In addition, the cross-sectional design of the survey restricts the ability to establish causality between variables. Since wearable usage may change over time, this type of study does not capture the dynamic changes in patterns of wearable use over time. We were also limited by the data already gathered by HINTS. Further research could explore reasons for nonuse or nonsharing of wearable data, and could examine additional questions pertaining to privacy, trust and design issues.

### Conclusion

This study highlights critical insights into the use and data-sharing behaviors associated with wearable health care technology. We have detailed the latest trends in the adoption and use of wearable devices, outlining the frequency and demographics of users. In addition, we explored how US adults engage in sharing data from wearable devices, including their willingness to share this information with health care providers and with family and friends. Our study noted major gaps between willingness to share health care wearable data, and the actual sharing behavior with providers. By identifying the sociodemographic, health and technological factors that influence both the use of wearables and the willingness to share data, our findings underscore the necessity for tailored interventions. Clinicians can enhance patient engagement by addressing the specific barriers identified, particularly among demographics with lower sharing rates. Policy makers should prioritize initiatives that promote accessibility and digital literacy, ensuring equitable benefits from wearable technologies. These targeted approaches can help bridge the gap between users’ willingness to share health data and their actual sharing behaviors, ultimately advancing the integration of wearable devices in personalized health care.

Looking forward, this study lays the groundwork for future research aimed at enhancing the design of digital health interventions and improving the integration of wearable technology in clinical practices. By continuing to investigate the evolving dynamics of user engagement and the barriers to data sharing, we can better leverage wearable technology to advance personalized health care and public health outcomes.

## Abbreviations

HINTS: Health Information National Trends Survey
IRB: institutional review board
NIH: National Institutes of Health
OR: odds ratio

## Data Availability

The Health Information National Trends Survey 6 (HINTS 6) data is publicly available and seeks to provide a comprehensive view of health communication trends among US adults. Conducted by the National Cancer Institute, this data collection focuses on how people access and use health-related information and behaviors. The complete datasets, including methodological reports and questionnaires, are accessible on the HINTS website [82].
